# Mitochondrial DNA Missense Mutations ChrMT: 8981A > G and ChrMT: 6268C > T Identified in a Caucasian Female with Myalgic Encephalomyelitis/Chronic Fatigue Syndrome (ME/CFS) Triggered by the Epstein–Barr Virus

**DOI:** 10.1155/2024/6475425

**Published:** 2024-05-09

**Authors:** Gaoyan G. Tang-Siegel, David W. Maughan, Milah B. Frownfelter, Alan R. Light

**Affiliations:** ^1^Department of Molecular Physiology and Biophysics, College of Medicine, University of Vermont, Burlington, VT, USA; ^2^Seattle Medical Associates, 1124 Columbia St. Suite 620, Seattle, WA, USA; ^3^Department of Anesthesiology, University of Utah, Salt Lake City, UT, USA

## Abstract

Myalgic encephalomyelitis/chronic fatigue syndrome (ME/CFS) is a multisystem disabling disease with unclear etiology and pathophysiology, whose typical symptoms include prolonged debilitating recovery from fatigue or postexertional malaise (PEM). Disrupted production of adenosine triphosphate (ATP), the intracellular energy that fuels cellular activity, is a cause for fatigue. Here, we present a long-term case of ME/CFS: a 75-year-old Caucasian female patient, whose symptoms of ME/CFS were clearly triggered by an acute infection of the Epstein–Barr virus 24 years ago (mononucleosis). Before then, the patient was a healthy professional woman. A recent DNA sequence analysis identified missense variants of mitochondrial respiratory chain enzymes, including *ATP6* (ChrMT: 8981A > G; Q152R) and *Cox1* (ChrMT: 6268C > T; A122V). Protein subunits ATP6 and Cox1 are encoded by mitochondrial DNA outside of the nucleus: the *Cox1* gene encodes subunit 1 of complex IV (CIV: cytochrome c oxidase) and the *ATP6* gene encodes subunit A of complex V (CV: ATP synthase). CIV and CV are the last two of five essential enzymes that perform the mitochondrial electron transport respiratory chain reaction to generate ATP. Further analysis of the blood sample using transmission electron microscopy demonstrated abnormal, circulating, extracellular mitochondria. These results indicate that the patient had dysfunctional mitochondria, which may contribute directly to her major symptoms, including PEM and neurological and cognitive changes. Furthermore, the identified variants of ATP6 (ChrMT: 8981A > G; Q152R) and Cox1 (ChrMT: 6268C > T; A122V), functioning at a later stage of mitochondrial ATP production, may play a role in the abnormality of the patient's mitochondria and the development of her ME/CFS symptoms.

## 1. Introduction

Myalgic encephalomyelitis (ME)/chronic fatigue syndrome (CFS) is a multisystemic disease [1, 2], which often severely disables the patient. ME/CFS affects millions of Americans, including many patients who have not been diagnosed and therefore have not received proper care, due to a lack of standard diagnostic tools and treatments and, primarily, a lack of understanding of the disease. The disease was identified in different age groups, which could be triggered by either a noticeable infection [3–6] or a noninfectious event, which includes trauma [7] or a pre-existing chronic condition, e.g., vasculitis [8]. The illness is typically characterized by slow recovery from fatigue physically and mentally, with additional broad symptoms including pain, sleep disturbance, neurological and cognitive changes, motor impairment, and altered immune and autonomic responses (dysautonomia). Among them, postexertional malaise (PEM) and memory/concentration problems (brain fog) are the primary debilitating symptoms [2].

Despite diverse triggers, ME/CFS appears to be triggered in most cases after a noticeable infection that was caused by a bacterium, a fungus, or more commonly a virus, including the highly disseminated Epstein–Barr virus (EBV) [3] and severe acute respiratory syndrome coronavirus 2 (SARS-CoV-2), which caused a devastating global pandemic [9]. The subsequent 35 million Americans suffering long coronavirus disease 19 (Long COVID, ) and similar symptoms [5, 6] shared with ME/CFS lend strong support to the hypothesis that ME/CFS is a postinfection syndrome, at least, in certain patient groups, and viral infections are the important triggers.

ME/CFS is featured with prolonged debilitating recovery from fatigue or PEM, as well as neurological disorders including dysautonomia, all of which are consistent with disruption of adenosine triphosphate (ATP) production. Blunted ATP production suggests dysfunctional mitochondria, as mitochondria are the intracellular organelles that produce most of our ATP through aerobic respiration. Mitochondria employ five critical respiratory chain enzymes (or complexes) for driving electron transportation to create a proton gradient for ATP production: complex I (CI, NADH: ubiquinone oxidoreductase), complex II (CII, succinate dehydrogenase), complex III (CIII, ubiquinol-cytochrome c oxidoreductase), complex IV (CIV, cytochrome c oxidase), and complex V (CV, ATP synthase) [10].

**Figure 1:**
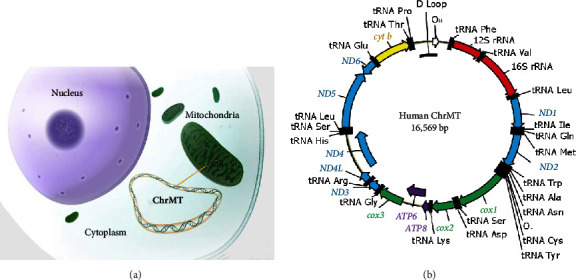
Chromosomes of mitochondria (ChrMT). Mitochondria in the cytoplasm of cells contain their own circular dsDNA (ChrMT). (a) ChrMT located outside of the nucleus. Offspring inherit ChrMT from their mothers. (b) Map of human ChrMT, derived from Shokolenko et al. [11]. *ND1*-*ND6* and *ND4L* encode subunits of CI; *Cox1*-*Cox3* encode subunits of CIV; *ATP6* and *ATP8* encode subunits of CV; and *cyt b* encodes CIlI. The ChrMT encodes 13 proteins, composed of complexes of CI, CIV, CV, and CIII.

These complexes are mainly encoded by mitochondrial chromosome DNA (ChrMT DNA, or mtDNA), which is circular DNA of ∼16,569 kbp, existing outside of the nucleus (Figure 1(a)). The ChrMT encodes 13 proteins [11], including *ND1*-*ND6* and *ND4L* that encode subunits of CI; *cox1*-*cox3* that encode subunits of CIV; *ATP6* and *ATP8* that encode subunits of CV; and *cyt b* that encodes CIII (Figure 1(b)). Unlike nuclear chromosomes following parental inheritance, mitochondrial chromosomes are hereditary following the maternal inheritance pattern. In addition, ChrMT is the location for oxidative phosphorylation (OXPHOS) to generate ATP, and consequently, the ChrMT DNA is 10–20 times more susceptible to oxidative damage and has been observed with high mutation rates [12]. The high mutation rate is due to (1) the abundance of reactive oxygen species (ROS) or free electrons generated through OXPHOS during ATP biosynthesis in the mitochondria [13] and (2) relatively less efficient mtDNA repair mechanisms compared with robust nuclear DNA damage repairs [12, 14].

Here, we present a female case of ME/CFS, with two identified variants of the ChrMT DNA-encoded proteins. Additional transmission electron microscopy (TEM) examination revealed abnormal extracellular mitochondria in the blood circulation. The evidence demonstrated in this case report lends strong support to the hypothesis that genetic and/or epigenetic mitochondrial abnormalities and dysfunction fundamentally contribute to the illness of ME/CFS.

## 2. Case Presentation

We present a 75-year-old Caucasian female patient with ME/CFS, who was diagnosed after an acute EBV infection (mononucleosis) at the age of 51. In December of 1999, she developed an acute flu-like illness: sore throat and fever for 10 days. Since then, the patient has experienced a sequence of global exacerbated illness followed by partial recovery, but never fully recovered. She presented mainly neurological and cognitive impairment, including fatigue, severe PEM, severe dysautonomia, unrefreshing sleep, widespread achiness and tenderness, sporadic dizziness with vertigo, severe orthostatic intolerance, and brain fog. After meeting with a few specialists, she was finally diagnosed with ME/CFS, based on the Fukuda criteria (CDC 1994 criteria) [1] in 2004, by a neuroscience professor and ME/CFS specialist, Dr. Benjamin H. Natelson, at the University of Medicine and Dentistry of New Jersey.

At the visit, the physical examination and laboratory tests showed normal. The vital signs were normal in the supine position. However, when the patient changed from the supine position to the standing position, her end-tidal CO_2_ (ETCO_2_) fell to 30 mmHg (normal range: 35–45 mmHg). In addition, her symptoms became worse when she voluntarily hyperventilated in 10 breaths.

In 2017, the patient visited a different ME/CFS specialist, Dr. Lucinda Bateman, in the Bateman Horne Center, Salt Lake City, UT, who made the same diagnosis based on the 2015 criteria of the Institute of Medicine (IOM, or National Academic of Medicine, NAM) [2]. In addition, the patient's symptoms of unrefreshing sleep, cognitive impairment, and severe orthostatic intolerance with 70% drop in pulse pressure from 44 mmHg to 13 mmHg in a 10-minute standing measurement, demonstrated by using the National Aeronautics and Space Administration Lean Test (NLT) [15], also made her meet the Canadian Consensus Criteria () as a ME/CFS patient.

Prior to her bout of EBV infection in 1999, the patient was a healthy, professional woman. After acquiring the infection, the patient has suffered from ME/CFS with severe dysautonomia, cognitive impairment, and widespread pain. The patient also maintained relatively high titers of EBV antibodies, including anticapsid antigen (anti-VCA) IgM/IgG and anti-early antigen (EA) IgG, despite the fact that she has been taking antiviral drugs, including valacyclovir and valganciclovir for long periods. The elevated anti-VCA and anti-EA antibodies indicated an acute phase of EBV infections, suggesting repeated re-infections (or lytic infections) of EBV, during multiple episodes of her “crash” times. A very recent blood test was performed in June 2023, which demonstrated anti-VCA IgG > 600.0 (standard range 0.0–17.9) and anti-EA IgG > 103.0 U/mL (standard range 0.0–8.9 U/mL).

The elevated anti-EBV antibodies of the patient, however, did not meet the diagnostic criteria for chronic active Epstein–Barr virus (CAEBV) disease, which requires extremely high antibody detection, including anti-EA IgG ≥ 160 U/mL [16]. In addition to antibody titers, simultaneous quantitative PCR analyses of her blood samples at the same time indicated relatively low viral DNA loads and her most current laboratory tests (June 2023) showed 248 IU/mL, significantly lower than 10,000 IU/mL, the diagnostic criteria for CAEBV [16]. Furthermore, the patient did not present any common symptoms of CAEBV, which are normally indicators of uncontrolled viral infiltrations of multiple organs, such as lymphadenopathy, splenomegaly, hepatitis, and pancytopenia [16, 17], except for a fever at the time when she was initially diagnosed with infectious mononucleosis in 1999.

Further ChrMT DNA sequencing analysis was performed in September 2023. Whole blood was collected using BD vacutainer tubes with K2 EDTA. The blood sample was centrifuged at 1, 200 × *g* for 10 min within 24 hours of collection. The middle puffy layer of cells, containing white blood cells (WBCs) and platelets, which appeared after the initial centrifugation, was used for cellular ChrMT DNA isolation. The top layer of plasma collected was then centrifuged at 2, 000 × *g* for 10 min to remove any contaminated cells [18]. After this second centrifugation, the top layer of plasma was collected and further centrifuged at 16, 000 × *g*, 4°C, for 30 min to pellet extracellular mitochondria for ChrMT DNA isolation and TEM analyses.

**Figure 2:**
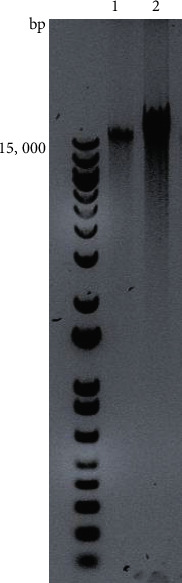
Mitochondrial chromosome DNA (ChrMT DNA) purification from the long-term ME/CFS female patient. 1. Extracellular ChrMT DNA isolated from the plasma; 2. cellular ChrMT DNA isolated from WBCs and platelets.

The DNA of plasma or cells was first isolated using the QIAamp DNA Kit (Qiagen, Germantown, MD) separately. Briefly, a pellet of the cells or plasma was resuspended in 180 *μ*L buffer ATL, mixed with Proteinase K, and incubated at 56°C overnight with occasional vortexing. The digested samples were added to 200 *μ*L buffer AL, incubated at 70°C for 10 min, and continued with the standard protocol using spin columns to isolate DNA. The isolated DNA was used for further selective purification and enrichment of mitochondrial ChrMT DNA using a REPLI-g Mitochondrial DNA Kit (Qiagen, Germantown, MD), based on the recommendation of the manufacturer. Briefly, the isolated DNA sample, from either the cells or the plasma pellet, was added to the REPLI-g reaction buffer mixed with REPLI-g human mitochondrial primers, preincubated at 75°C for 5 min, followed by adding DNA polymerase, incubating at 33°C for 8 hours, and then inactivation of the enzyme at 65°C for 3 min [19, 20]. The ChrMT DNA was isolated from the cells (including WBCs/platelets) and the plasma (Figure 2) and stored at −80°C before the sequencing analysis.

Sequencing analysis was performed using primers, and the amplified fragments were cloned into TOPO vectors (Invitrogen, Waltham, MA) for Sanger sequencing. An amplicon of 3,006 base pairs containing the *cox1* gene was generated using the primer set of 3734_For: 5′-aagtcaccctagccatcattcta-3′ and 6739_Rev: 5′-gatatcatagctcagaccatacc-3′ [21]. Another fragment of 2,710-base-pair amplicon containing the *ATP6* gene was generated using the primer set of 6511_For: 5′-ctgctggcatcactatactacta-3′ and 9220_Rev: 5′-gattggtgggtcattatgtgttg-3′. [21] The isolated ChrMT DNA from either the plasma or cells revealed variants of ChrMT, including ATP6 (ChrMT: 8981A > G; Q152R) and Cox1 (ChrMT: 6268C > T; A122V). Sequencing was performed by Eurofins Genomics (Louisville, KY).

**Figure 3:**
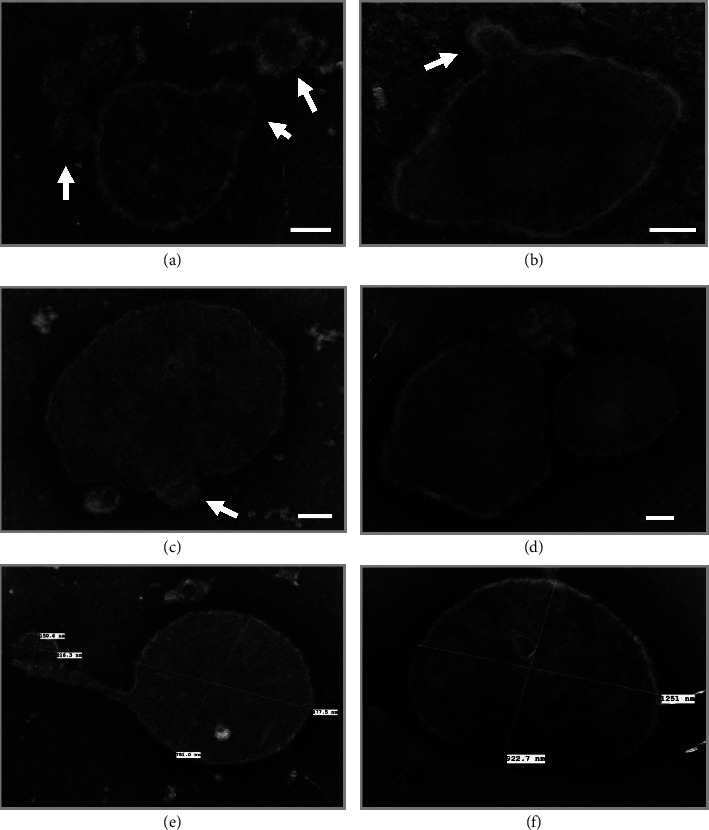
Methylamine tungstate, negatively stained, whole-mount TEM analysis of mitochondria (mt) isolated from the patient's plasma. Mitochondria with different shapes and sizes, ranging from ∼400 nm (Panel (a)) to over 1 *µ*m (Panel (f)), were identified. Some mitochondria are attached to “vesicle-like” structures protruding from the membrane (∼100 nm in diameter), pointed by white arrows in Panels (a–c). Scale bar: 100 nm.

The mitochondria isolated from the patient's plasma were further analyzed using methylamine tungstate (Nano-W, Nanoprobes, Yaphank, NY) negatively stained, whole-mount TEM [22, 23]. Mitochondria were identified with altered shapes, including irregular shapes (Figure 3), and different sizes, ranging from 400 nm to over 1 *μ*m. Some mitochondria appeared to “protrude” vesicle-like structures (approximately 100 nm in diameter) from the membrane (Figure 3).

## 3. Discussion

The 75-year-old female patient presented in this study is a long-term ME/CFS patient, who was diagnosed with ME/CFS two decades ago after an acute infection with EBV. Over the past 20 years, she has maintained relatively high levels of anti-VCA IgG and anti-EA IgG, as well as positive detection of viral replication, suggesting repeated, lytic viral infections, despite the fact that she was taking antiviral drugs.

However, her antibody titers and levels of viral loads were lower than the diagnostic criteria for CAEBV [16]. The absence of common symptoms that indicate uncontrolled viral infections of multiple organs further eliminated the possibility of CAEBV [16, 17]. The elevated antibody titers and the presence of viral DNA in the blood could be also due to the viral genome-encoded virologs, which are identified from EBV, interfering with the host's apoptosis and elimination of infected cells [24, 25].

The dsDNA EBV is a highly disseminated virus with over 95% positive serological detection, even in those under the age of 25 [26], but in this patient, her first devastating acute infection was identified when she was 51. The EBV infects and replicates in the oral epithelial cells and B lymphocytes [27], transmitted among individuals through saliva [28, 29]. The infected B lymphocytes become the major viral reservoirs and play important roles in viral life-long persistence in infected hosts. EBV infections not only lead to benign mononucleosis [30] but also cause malignancy, including the well-recognized nasopharyngeal carcinoma [31] and Burkitt lymphoma [32, 33]. Strikingly, EBV DNA replication has been found to be significantly more active in ME/CFS patients than in healthy individuals [3], including the current patient. This female patient showed significantly high anti-EA IgG antibody production (the titer was 6–10 times higher than the normal detection range, including the latest two tests in the past year), concomitant with her typical ME/CFS symptoms.

**Figure 4:**
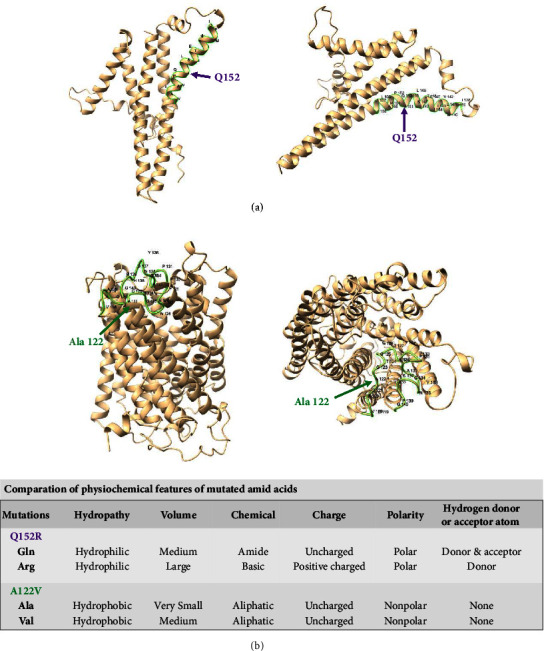
Structural localization of identified ChrMT DNA mutations in proteins and analyses of physiochemical features of mutated amino acids. (a) Complex V subunit A and ChrMT: 8981A > G (ATP6: Q152R, pointed by purple arrows). The mutation is located in one transmembrane helical structure (138–158, highlighted in green). The mutation from Gln to Arg changes certain physiochemical features. (b) Complex IV subunit 1 and ChrMT: 6268C > T (Cox1: A122V, pointed by green arrows). The mutation from Ala to Val is located in a topological domain (118–140, highlighted in green) and appears to cause less consequence on the protein structure and/or function. Structure predictions were prepared using AlphaFold [34].

The ChrMT DNA and protein sequence analysis revealed at least two variants, including ATP6 (ChrMT: 8981A > G Q152R) and Cox1 (ChrMT: 6268C > T A122V). Further analyses of these variants based on the structural location of the mutation within the protein [34] and physiochemical comparison of the mutated amino acids are illustrated in Figure 4. The Q152R mutation of the ATP6 protein unit is located in one of six transmembrane helical structure motifs: 6–26, 68–88, 97–117, 138–158 (as labelled in green in Figure 4(a)), 164–184, and 189–209 (). The structural analysis of complex V subunit A (encoded by the *ATP6* gene, Figure 4(a)) indicated that the mutation from “Gln” to “Arg” is located in a critical helical structure of the protein unit. The amino acid “Arg” presents different physiochemistry compared with “Gln,” including volumes, chemical features, charges, and whether they are donors or acceptors (Figure 4). These physiochemical alterations may eventually lead to structural and/or functional changes. However, the mutation of ChrMT:6268C > T; Cox1: A122V is located in a topological domain of complex IV subunit 1 (encoded by the *cox1* gene, Figure 4(b); ). Except for different volumes for amino acids “Ala” and “Val,” the similar physiochemical features shared by these two amino acids (Figure 4) suggest that the A122V mutation alone is less likely to cause structural and/or functional changes (Figure 4(b)), which, however, requires further investigation, as the Cox1 structure gene mutation is extremely rare [35].

Notably, pathogenic ATP6 variants, e.g., ChrMT: 8993T > G, have been linked to mitochondrial and neurological disorders [36]. Multiple pathogenic ATP6 mutations have been identified up to December 2023, based on the database searching using (Table 1). However, up to December 2023, none of the Cox1 variants have been reported, based on the database searching using . Not only Cox1 but also mutations that occurred in Cox structural genes, encoded by either mtDNA or nuclear DNA, are extremely rare [35]; a few identified Cox deficiency syndromes are due to mutations in the assembly factors [35]. However, different Cox1 deficiency model systems, established using animals including *Saccharomyces cerevisiae*, *Drosophila melanogaster,* and *Mus musculus*, have demonstrated diverse phenotypes, including Leber hereditary optic neuropathy (LHON), acquired idiopathic sideroblastic anemia (AISA), ataxia, hypotonia, and epilepsy [37].

To our knowledge, the ChrMT: 6268C > T; Cox1: A122V variant, which we identified here, is the first reported mutation of the *cox1* gene. Nevertheless, complex CIV achieves its function in the electron transport chain (ETC) by forming supercomplexes (CICIII_2_CIV and CIII_2_CIV) with complexes CI and/or CIII [10], suggesting that dysfunctional CIV will also affect the functions of upstream enzyme complexes, which was supported by an *in vitro* model [38]. Therefore, mutations that occurred in complexes CIV and CV, which are functioning at the later stage of the ETC, will have more progressive effects on ATP production and mitochondrial functions. We proposed that the mutations we have identified and characterized here may have potentially predisposed this patient to develop abnormal mitochondria, as observed, which could have contributed to her major symptoms of ME/CFS: debilitating PEM and neurological and cognitive alterations.

Interestingly, an extensive study performed by Billing-Ross et al. investigated the correlation between ChrMT DNA variants and ME/CFS based on 193 cases *versus* 196 control individuals and indicated that ME/CFS patients with certain mitochondrial haplogroups or point mutations at certain positions (ChrMT:150, ChrMT:930, ChrMT:1719, ChrMT:3010, ChrMT:5147, ChrMT:16093, ChrMT:16223, and ChrMT:16519) are more likely to develop certain symptoms [39]. However, the ChrMT:8981 and ChrMT:6268 mutations, which we identified in this patient, were not on their list. Furthermore, the study performed by Billing-Ross et al. did not identify any ChrMT variants, which would potentially increase the susceptibility of an individual to develop ME/CFS [39]. Given these findings, we hypothesize that the point mutations of ChrMT identified in this patient might occur later in her life randomly, due to accumulated mitochondrial stress, including viral exposure and infection. Consistently, there are studies convincing a strong correlation between ME/CFS and mitochondrial dysfunction [40, 41].

Together with the evidence of low viral replications identified from the blood sampled at different times over a two-decade period in this ME/CFS patient, our data suggest that the ChrMT mutations identified in this patient may have initially predisposed her to an enhanced vulnerability to the consequences of viral exposure, which eventually led to her susceptibility to developing her major symptoms of ME/CFS, including brain fog and PEM. The accumulated evidence suggests that a consequently combined effect of malfunctioning mitochondria and compromised immunity promoted the development of severe ME/CFS in this patient, although we do not know at this time whether the mutations were carried from the patient's mother or mutations occurred after her birth.

As we mentioned above, the abundance of ROS in the mitochondria [13] and nonequivalent DNA repair mechanisms [12, 14] cause ChrMT DNA to be over 10 times more susceptible to oxidative damage and DNA mutation than nuclear DNA [12]. It is possible that the ChrMT DNA mutations occurred after the patient's life-time exposure to viruses. Viruses tend to hijack host cells and reprogram energy production [24, 25, 42]. However, the patient experienced her most prominent neurological and cognitive changes later in her life, including severe dysautonomia, unrefreshing sleep, sporadic dizziness with vertigo, severe orthostatic intolerance, and brain fog. The timing and severity of her symptoms were not limited to her immune cells, if the mutations did occur later in her life, which suggests that ChrMT DNA sequencing analyses of family members of the patient would be an important further step to understand the individual and combined role of viral infections, mitochondrial ChrMT DNA mutations, and mitochondrial malfunction in the development of postinfection syndromes with profound disabling fatigue featured in the typical case of ME/CFS reported here.

Circulating mitochondria in the blood have been the subjects of recent investigations [18, 43]; some focused on their possible contributions to diseases [44], and others focused on their potential uses as biomarkers [45]. Although double-membrane mitochondria are organelles of our cells, evolutionarily, these power plants of eukaryotic cells are considered to be originally derived from aerobic prokaryotes [25, 46]. This endosymbiotic theory [46] has been favored and supported by similarities shared with Gram-negative bacteria, which also have outer and inner membranes, as well as some similar membrane-anchored proteins [47–49].

In this case of ME/CFS, we observed that free, extracellular mitochondria appeared to “protrude” or “secrete” vesicle-like structures (Figure 3), like those secreted from Gram-negative bacteria in stressful conditions. Recently, the essential roles that mitochondria may play during the viral invasion of the host have been recognized [25]. It is likely that the abnormal appearance of the free mitochondria we observed in this patient is a consequence of malfunctional mitochondria responding to viral exposures. The causes and sequelae of large numbers of extracellular, abnormal mitochondria in the blood circulation of this ME/CFS patient, and of other patients with ME/CFS-like, postinfection syndromes, require further investigations.

Mitochondria are also important antiviral responders in host immunity and are exposed to unique *in vivo* environments, which were demonstrated by increasing evidence [25]. Due to the important roles that mitochondria play against viral infections, viruses also change to adapt themselves better *in vivo*, by generating large populations of viral genome-encoded virologs [50], which include mimicking the host's enzymes and interfering with the mitochondrial tricarboxylic acid (TCA) cycle and ETC reaction of host cells to inhibit the apoptosis of infected cells. The presence of virologs will further increase viral persistence and the stress and vulnerability of the mitochondria during viral infections [24, 25].

In summary, the ME/CFS case presented here suggests that dysfunctional mitochondria predispose individuals to develop ME/CFS after viral infections. Mitochondrion-associated predispositions that promote patients to develop ME/CFS could be either (1) gene variants inherited at the time of birth, (2) epigenetic modifications, or (3) DNA mutations that occurred after viral exposures and/or infections. This conclusive hypothesis is supported by the fact that mitochondrial DNA is both highly susceptible to oxidative damage and mutates at a higher rate than nuclear DNA.

## Figures and Tables

**Table 1:** Identified pathogenic *ATP6* variants.

Variation location	Genes	Conditions	Clinical significance (last reviewed)	Review status
NC_012920.1 (MT-ATP6): m.8783G > A	MT-ATP6	Leigh syndrome, Leber optic atrophy	Pathogenic/likely pathogenic (May 4, 2022)	Criteria provided, multiple submitters, no conflicts

NC_012920.1 (MT-ATP6): m.8993_8994inv	MT-ATP6	NARP syndrome	Pathogenic (October 17, 2019)	Criteria provided, single submitter

NC_012920.1: m.8993T > C	MT-ATP6	Mitochondrial disease	Pathogenic (February 17, 2021)	Reviewed by an expert panel
				FDA-recognized database

NC_012920.1: m.8993T > G	MT-ATP6	Mitochondrial disease	Pathogenic (March 22, 2021)	Reviewed by an expert panel
				FDA-recognized database

NC_012920.1: m.9176T > C	MT-ATP6	Mitochondrial disease	Pathogenic (June 30, 2022)	Reviewed by an expert panel
				FDA-recognized database

NC_012920.1: m.9185T > C	MT-ATP6	Mitochondrial disease	Pathogenic (June 30, 2022)	Reviewed by an expert panel
				FDA-recognized database

^*∗*^Based on the searching results of the database:in December 2023.

## Data Availability

All the important data are included in this article, and extra information may be provided upon request.

## Additional Points

*Key Clinical Message*. ME/CFS is a multisystem disabling disease with unclear etiology and population selection mechanisms. Viral infections are known to be important triggers in certain patient groups. We report one female case, triggered by an infection of the Epstein–Barr virus, which was identified with mitochondrial DNA variants that were potentially linked to her illness.
